# Correlation analysis between disease severity and inflammation-related parameters in patients with COVID-19: a retrospective study

**DOI:** 10.1186/s12879-020-05681-5

**Published:** 2020-12-21

**Authors:** Jing Gong, Hui Dong, Qing-Song Xia, Zhao-yi Huang, Ding-kun Wang, Yan Zhao, Wen-hua Liu, Sheng-hao Tu, Ming-min Zhang, Qi Wang, Fu-er Lu

**Affiliations:** 1 Department of Integrated Traditional Chinese and Western Medicine, Tongji Hospital, Tongji Medical College, Huazhong University of Science and Technology, Wuhan, China; 2 Institute of Integrated Traditional Chinese and Western Medicine, Tongji Hospital, Tongji Medical College, Huazhong University of Science and Technology, Wuhan, China; 3 Clinical Research Center, Tongji Hospital, Tongji Medical College, Huazhong University of Science and Technology, Wuhan, China

**Keywords:** COVID-19, Inflammation, Disease severity, Blood cell count, Cytokine

## Abstract

**Background:**

COVID-19 is highly contagious, and the crude mortality rate could reach 49% in critical patients. Inflammation concerns on disease progression. This study analyzed blood inflammation indicators among mild, severe and critical patients, helping to identify severe or critical patients early.

**Methods:**

In this cross-sectional study, 100 patients were included and divided into mild, severe or critical groups according to disease condition. Correlation of peripheral blood inflammation-related indicators with disease criticality was analyzed. Cut-off values for critically ill patients were speculated through the ROC curve.

**Results:**

Significantly, disease severity was associated with age (*R* = -0.564, *P* < 0.001), interleukin-2 receptor (IL2R) (*R* = -0.534, *P* < 0.001), interleukin-6 (IL-6) (*R* = -0.535, *P* < 0.001), interleukin-8 (IL-8) (*R* = -0.308, *P* < 0.001), interleukin-10 (IL-10) (*R* = -0.422, *P* < 0.001), tumor necrosis factor α (TNFα) (*R* = -0.322, *P* < 0.001), C-reactive protein (CRP) (*R* = -0.604, *P* < 0.001), ferroprotein (*R* = -0.508, *P* < 0.001), procalcitonin (*R* = -0.650, *P* < 0.001), white cell counts (WBC) (*R* = -0.54, *P* < 0.001), lymphocyte counts (LC) (*R* = 0.56, *P* < 0.001), neutrophil count (NC) (*R* = -0.585, *P* < 0.001) and eosinophil counts (EC) (*R* = 0.299, *P* < 0.001). With IL2R > 793.5 U/mL or CRP > 30.7 ng/mL, the progress of COVID-19 to critical stage should be closely observed and possibly prevented.

**Conclusions:**

Inflammation is closely related to severity of COVID-19, and IL-6 and TNFα might be promising therapeutic targets.

## Background

Global countries are going through a battle between humans and the virus named SARS-CoV-2 [1]. Phylogenetic analysis of viral complete genome revealed that the novel virus was most similar to a group of SARS-like coronaviruses (genus Betacoronavirus, subgenus Sarbecovirus) [2, 3]. Patients infected with SARS-CoV-2 have a series of clinical manifestations, including fever, cough, myalgia or fatigue, dyspnea, even acute respiratory distress syndrome (ARDS), acute cardiac injury and secondary infection, and a lot of severe patients had to been admitted to the intensive care unit (ICU) [4, 5].

Clinically, most individual patients showed the changes of neutrophil count (NC), D-dimer, blood urea nitrogen, and creatinine levels and lymphocyte counts (LC) [6] besides the positive viral nucleic acid analysis and the representative pulmonary CT findings (bilateral distribution of patchy shadows and ground glass opacity). Increased inflammation-related biomarkers were found in COVID-19 patients, including interleukin-6 (IL-6), C-reactive protein (CRP), ferroprotein, and so on [7]. However, the correlation between the inflammatory markers and the disease severity is still not completely clear. Therefore, this study retrospectively analyzed blood inflammation indicators among mild, severe and critical patients, which may help to identify severe or critical patients early and perform clinical intervention early.

## Methods

### Patients

This was a retrospective observational study. Laboratory data of patients diagnosed with COVID-19 and received peripheral blood cytokines tests were collected from February 10, 2020 to February 15, 2020 in Tongji Hospital, Tongji Medical College of Huazhong university of Science and Technology. Collected data included disease severity, age, gender, cytokines, inflammatory parameters and blood cell counts. As listed below, the diagnosis of severe or critical patients were depending on “New Coronavirus-Infected Pneumonia” Severe and Critical Diagnosis and Treatment Program (Second trial version) formulated by the National Health Commission of China. *Standard of severe or critical patients:*

Severe patients should have any of the following conditions:

1. Respiratory distress, breathing rate ≥ 30 times / minute;
2. Under the resting state, the oxygen saturation ≤ 93%;
3. Oxygen partial pressure (PaO2)/oxygen concentration (FiO2) in arterial blood ≤300 mmHg.
4. > 50% lung imaging progress in the short term.

Critical patients should have any of the following conditions:

1. Respiratory failure occurs and mechanical ventilation required;
2. Shock occurs;
3. Combining other organ failure and requiring treatment in ICU.

### Inflammation-related biomarkers

All tests were completed in the clinical laboratory in Tongji Hospital. Interleukin-1β (IL-1β), interleukin-2 receptor (IL2R), interleukin-8 (IL-8), interleukin-10 (IL-10) and tumor necrosis factor α (TNFα) were detected by Siemens chemiluminescence method, and IL-6 were detected by Roche electrochemiluminescence method according to the manufacturer’s instruction. The ultrasensitive CRP regent was provided by Nippon Denkasei Co., Ltd., and CRP was detected by immunoturbidimetry method. Ferroprotein was detected by Roche granule-enhanced immune turbidimetry. Procalcitonin (PCT) was detected by Roche electrochemiluminescence method. ESR was measured by Westergren’s international standard method. Peripheral blood cell was detected by fluorescence staining flow cytometry. We then analyzed the differences of white blood cell (WBC), NC, LC and eosinophils (EC) among three groups.

### Statistical analysis

For data of normal distribution (IL2R, ESR, ferroprotein, WBC and NC), comparisons among critical, severe and mild groups were analyzed by ANOVA analysis. The pairwise comparison between groups was performed using the Bonferroni test when the variance is uniform, and Dunnett’s T3 test was used when the variance is not uniform. For non-normal distribution (CRP and LC), the data were conversed using square root, followed by ANOVA analysis and pairwise comparison. With data below the detectable limit, including IL-1β, IL-6, IL-8, IL-10, TNFα, PCT, and EC, there were no accurate values, and the data were ranked referring to the reference range and value rank (Table S1); then non-parametric Kruskal-Wallis test were performed. In correlation analysis, Spearman correlation coefficient or Kendall’s tau-b correlation coefficient was used according to the data. Unconditional logistic regression model was used to find the associated factors for critical illness.

To find out cut-off points of inflammatory parameters for critical patients, receiver operating characteristic (ROC) analyses were administrated. AUC was used for prediction strength, and optimum cut-off points were chosen using Youden’s index. Data were analyzed using SPSS 20.0.

### Ethics approval

This study was approved by the ethical committee of Tongji Hospital, Tongji Medical College, Huazhong University of Science and Technology. Because of the infectivity and the exploration urgency of this disease, written informed consent was waived by the ethical commission.

## Results

**Fig. 1:**
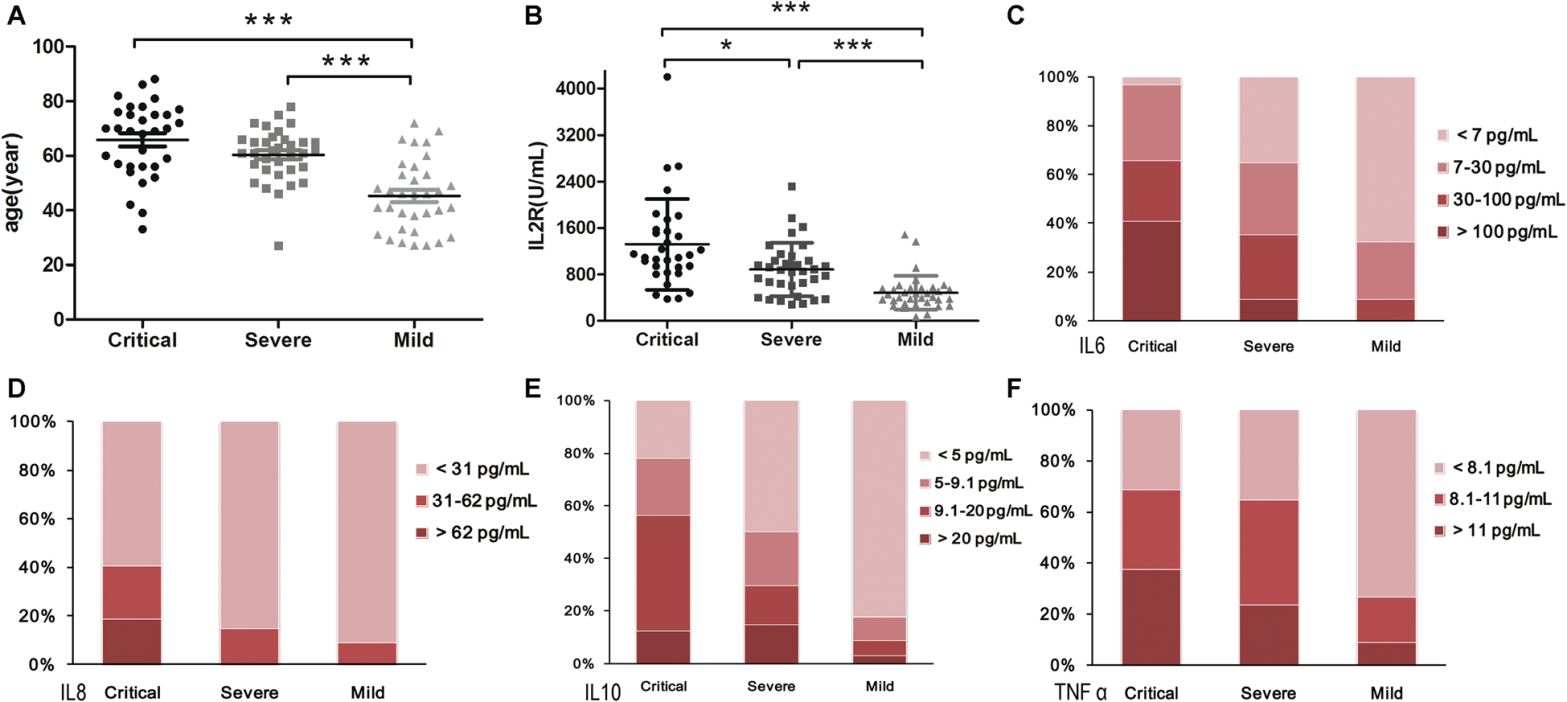
Comparison of age and cytokines among mild, severe and critical patients with COVID-19. **a** age; **b** IL2R; **c** distribution comparison of IL-6; **d** distribution comparison of IL-8; **e** distribution comparison of IL-10; **f** distribution comparison of TNFα. Many examination data about IL-6, IL-8, IL-10 and TNFα were below the lowest testing limit, different ranks were set according to data distribution and reference range, and data distribution among different group were compared (**c**-**f**). Data are presented as mean ± standard error or percentage of distribution range. IL2R, interleukin-2 receptor; IL6, interleukin-6; IL8, interleukin-8; IL10, interleukin-10; TNFα, tumor necrosis factor α.*P < 0.05; **P < 0.05; ***P < 0.001

**Fig. 2:**
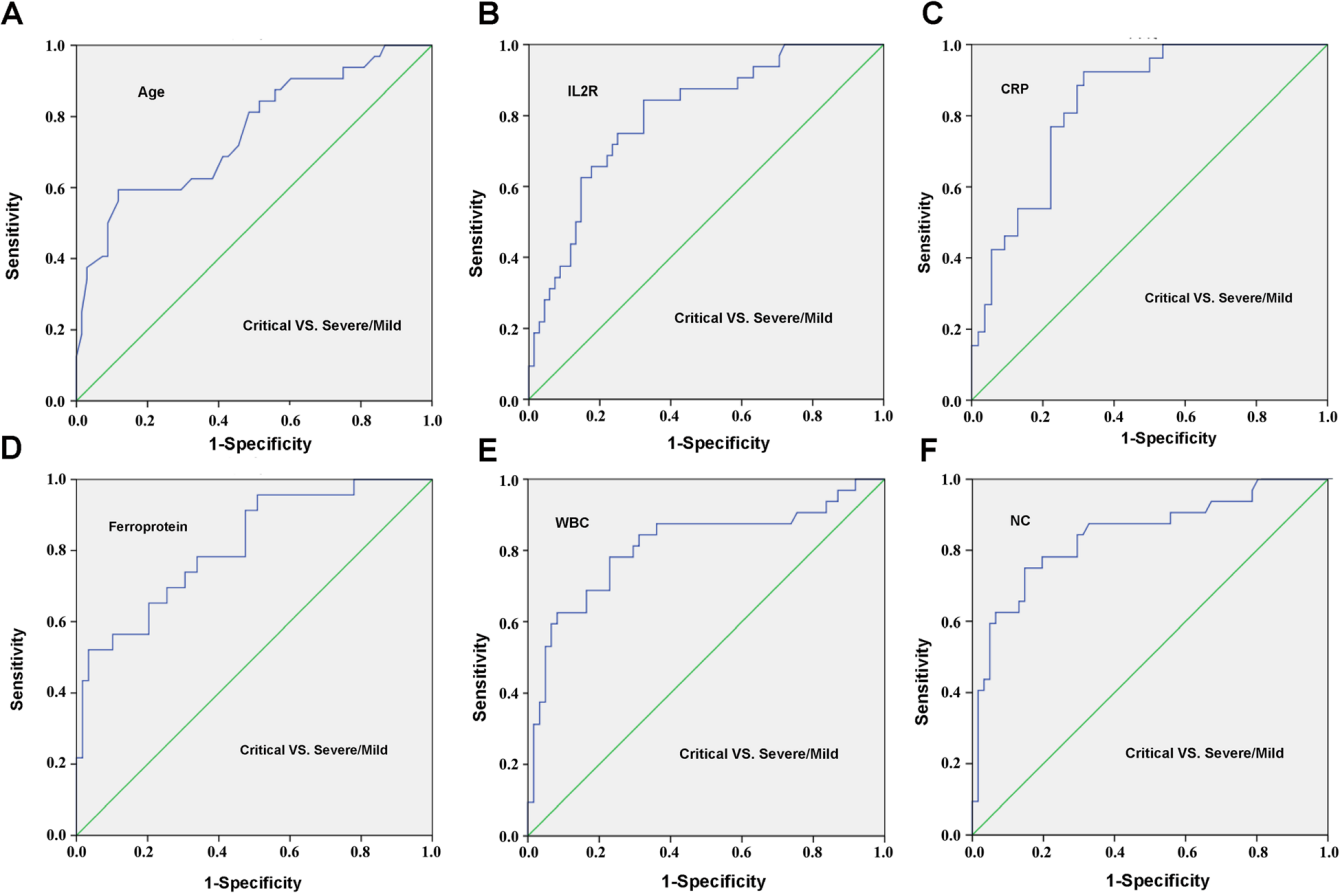
ROC curve of age and inflammatory parameters for critical illness of COVID-19. **a** age; **b** IL2R; **c** CRP; **d** ferroprotein; **e** WBC; **f** NC. IL2R, interleukin-2 receptor; CRP, C-reactive protein; WBC, white cell counts; NC, neutrophil count. AUC, 95% CI and *P* values are shown in the figure

Among the 100 included patients, 34 patients belong to mild group, 34 were severe, and 32 were critical. The average age was 57.02 years, and 59% patients were male. As shown in Fig. 1a, the age of mild group (mean ± SD: 45.29 ± 13.08 years) was significantly different from that of severe patients (mean ± SD: 60.41 ± 9.80 years) or critical patients (mean ± SD: 65.88 ± 13.61 years), and there was no significant difference between severe and critical patients. To better distinguish the critical illness, the ROC curve of age was administrated and listed in Fig. 2a (AUC = 0.755, *P* < 0.001), and the best cut-off point of age for critical illness was 67.5 years with a specificity of 88.2% and a sensitivity of 59.4%. The optimum cut-off point of age for non-mild illness were 49.5 years (AUC = 0.838, 89.4% sensitivity, 67.6% specificity, *P* < 0.001, Fig. S1). The mean IL2R level was 1317.31 U/mL in critical group, 885.09 U/mL in severe group and 486.44 U/mL in mild group, with significant differences among three groups (*P* < 0.001, Fig. 1b). The ROC curve of IL2R (AUC = 0.8, P < 0.001) was shown in Fig. 2b, the best cut-off point for critical illness was 793.5 U/mL with a specificity of 67.6% and a sensitivity of 84.4%, and the optimum cut-off point for non-mild illness was 621.5 U/mL (AUC = 0.844, 80.3% sensitivity, 88.2% specificity, *P* < 0.001, Fig. S1).

Because some values were below the detectable limit, the test parameters were graded according to reference range and rank order regarding IL-6, IL-8, IL-10 and TNFα (Table S1). As shown in Fig. 1c, the IL-6 levels were significantly different among three groups, and all IL-6 concentrations were < 100 pg/mL in mild patients. Although the IL-8 reference range was < 62 pg/mL, the IL-8 levels in most patients were < 31 pg/mL, three ranked grades were set, namely, < 31 pg/mL, 31–62 pg/mL and > 62 pg/mL. Figure 1d showed that all IL-8 levels in mild and severe patients were within the reference range (< 62 pg/mL), and there were significant differences between critical and severe patients or critical and mild group (*P* < 0.05). With regard to IL-10 and TNFα, there were significant differences between mild and severe, as well as mild and critical patients, and no significant difference was found between severe and critical group (Fig. 1e-f). With regard to cytokine IL-1β, no significant difference was found among three groups, and the data were not shown.

**Fig. 3:**
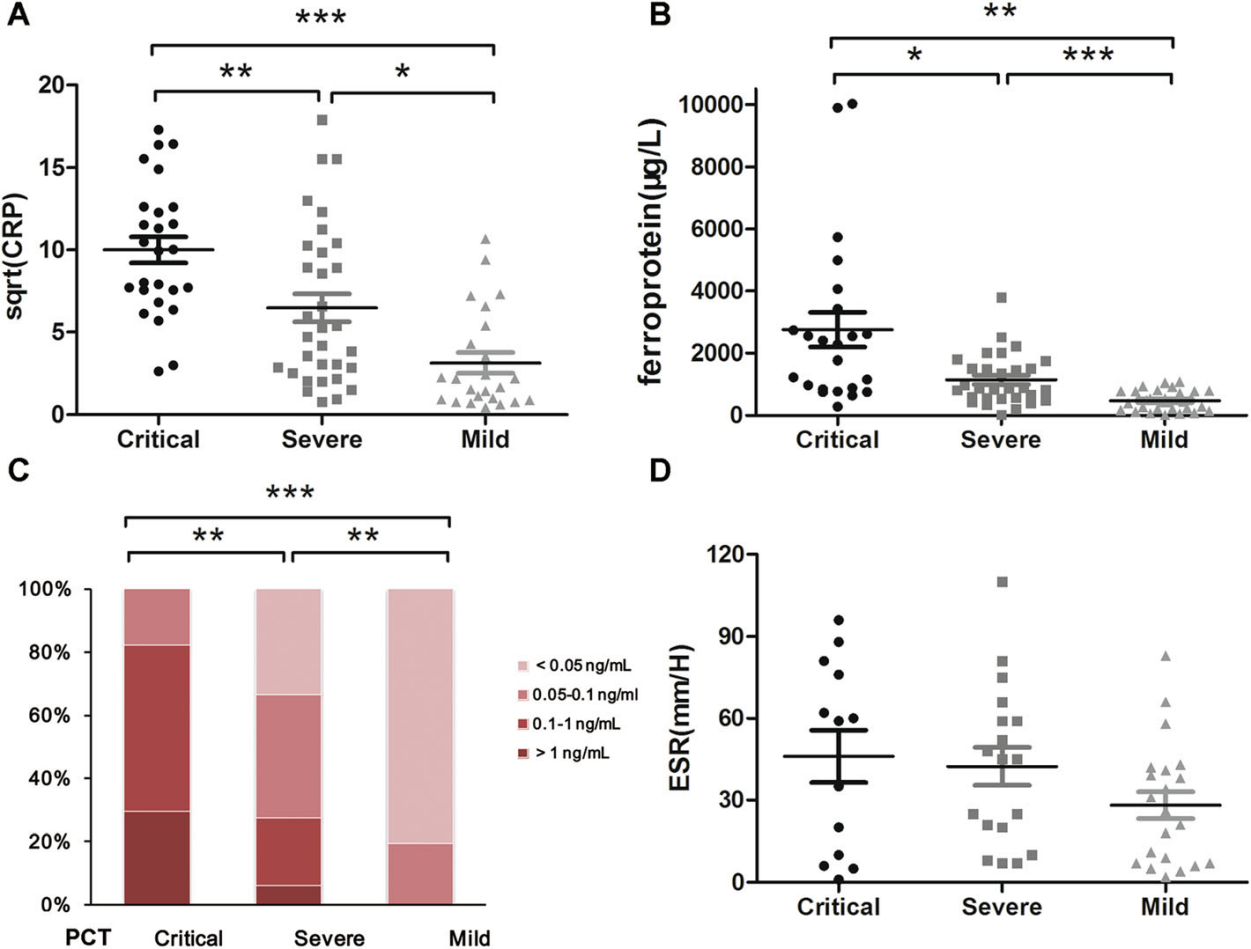
Comparison of inflammatory parametes among mild, severe and critical patients with COVID-19. **a** sqrt (CRP); **b** ferroprotein; **c** PCT; **d** ESR. CRP, C-reactive protein; PCT, procalcitonin; ESR, erythrocyte sedimentation rate. Data are presented as mean ± standard error or percentage. *P < 0.05; **P < 0.05; ***P < 0.001

In addition, levels of CRP, ferroprotein and PCT were statistically different among mild, severe and critical patients (Fig. 3a-c). All PCT levels were < 0.1 ng/mL in the mild patients, while PCT concentrations in all critical patients were > 0.05 ng/mL. ROC curve of CRP (AUC = 0.838, *P* < 0.001, Fig. 2c) suggested the best cut-off point was 30.7 ng/mL with a specificity of 92.3% and a sensitivity of 68.5%. The best cut-off point for non-mild illness on CRP was 6.2 ng/mL (AUC = 0.802, 85.7% sensitivity, 66.7% specificity, *P* < 0.001, Fig. S1). The average values of ferroprotein were 2753.87 μg/L in critical group, 1147.55 μg/L in severe group and 475.85 μg/L in mild group, respectively. ROC curve of ferroprotein (AUC = 0.814, *P* < 0.001, Fig. 2d) indicated the best cut-off point for critical illness was 2252 μg/L with a specificity of 96.6% and a sensitivity of 52.2%. The best cut-off point for non-mild illness on ferroprotein was 815.9 ng/mL (AUC = 0.837, 69.8% sensitivity, 86.2% specificity, *P* < 0.001, Fig. S1). There was no statistical difference on ESR among three groups (Fig. 3d).

**Fig. 4:**
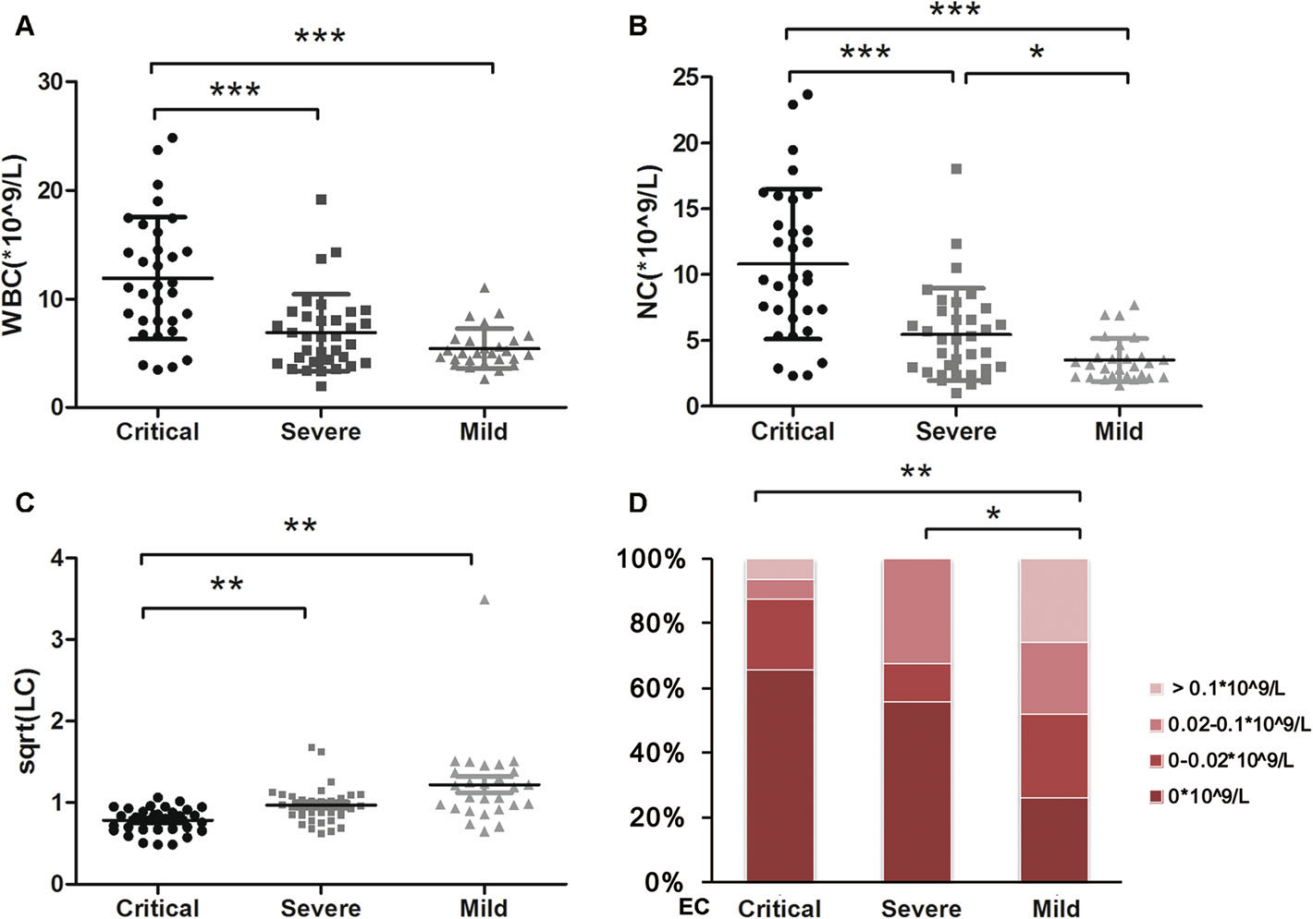
Comparison of blood cell counts among mild, severe and critical patients with COVID-19. Data are presented as mean ± standard error or percentage. **a** WBC; b NC; **c** sqrt (LC); **d** EC. WBC, white cell counts; NC, neutrophil count; LC, lymphocyte counts; EC, eosinophil counts. *P < 0.05; **P < 0.05; ***P < 0.001

In peripheral blood cell analysis, there were significant differences in WBC count between the critical and severe groups, as well as critical and mild groups, and no significance was found between the mild and severe groups (Fig. 4a). ROC curve of WBC (AUC =0.813, *P* < 0.001, Fig. 2e) suggested the best cut-off point for critical illness was 7.92*10^9/L with 77% specificity and 78.1% sensitivity, and the optimum cut-off point for non-mild illness was 6.445*10^9/L (AUC = 0.734, 69.7% sensitivity, 81.5% specificity, *P* < 0.001, Fig. S1). If the detected values of WBC are above the upper reference limit, it often indicates infection. Because WBC count is influenced by therapy, clinically the upper limit of reference range of WBC, namely 9.5*10^9/L, seems more meaningful.

NC was significantly different among the three groups (Fig. 4b), and the average was 10.80*10^9/L in critical group, 5.47*10^9/L in severe group and 3.53 *10^9/L in mild group. ROC curve of NC (AUC = 0.844, P < 0.001, Fig. 2f) indicated the best cut-off point was 7.305*10^9/L with 85.2% specificity and 75% sensitivity, and the optimum cut-off point for non-mild illness was 5.335*10^9/L (AUC = 0.734, 65.2% sensitivity, 88.9% specificity, *P* < 0.001, Fig. S1). There was significantly difference in LC between critical and severe group or critical and mild group (Fig. 4c). Many patients in each group had a decreased level of EC, and 0 EC was detected in 65.63% patients in critical group, 55.88% in severe group, and 25.93% in mild group. There were significant differences between severe and mild or critical and mild patients (Fig. 4d).

Table S2 showed correlation analysis results between the indicators with disease severity. Significant correlations were found about age, IL2R, IL-6, IL-8, IL-10, TNFα, CRP, ferroprotein, PCT, LC, NC, and EC. PCT (*R* = -0.650), CRP (*R* = -0.604), NC (*R* = -0.585), age (*R* = -0.564), LC (*R* = 0.56), IL-6 (R = -0.535), IL2R (*R* = -0.534) and ferroprotein (*R* = -0.508) were highly correlated (Table S2). There was no significant correlation on IL-1β, gender and ESR. Through logistic regression analysis, higher odds of critical illness was found in patients with high IL1β, IL6, IL10 and WBC levels, as well as low LC (Table S3).

## Discussion

COVID-19 is highly contagious, and about 80.9% patients are reported to be mildly to moderately severe [1], and with a better prognosis. However, for patients developing into severe or critical phase, the mortality rate was significantly increased, and crude mortality rate reached 49% in critical patients [1]. It plays key roles to identify severe and critical patients even earlier, which might improve the recovery rate and reduce mortality.

Clinically, many COVID-19 patients appeared short-term progressive aggravation. Scholars speculated that “inflammatory storms” occurred in patients, namely, overreaction of cytokines. In a preprint article in medrxiv, the lymphocyte subsets and cytokines of 123 patients (102 mild and 21 severe patients) were analyzed, and the researchers found the numbers of CD4+ T cells and CD8+ T cells decreased, and the levels of IL-6 and IL-10 increased in severe cases [8]. In this study, a retrospective analysis was conducted about cytokines and other inflammatory indicators of 100 patients (including 66 severe or critical patients), and more indicators were expected to better identify critical patients and help clinical decision-making.

In accordance with studies from S-XW and JL [8, 9], we also found that the levels of IL-6 and IL-10 were associated with the severity of COVID-19. TNFα concentration, NC count and LC count were also found correlated with disease severity, which are similar to C-LH’s study [5]. In addition, IL2R, ferroprotein, PCT, and EC counts were also related to the disease severity. Besides, we also found the IL-6 levels in mild patients were lower than 100 pg/mL. For eight patients who were deceased in critical group, IL-6 level was > 100 pg/mL in three persons. IL-6 > 100 pg/mL might represent the emergence of “inflammatory storm”. IL-8 levels in mild and severe patients were normal, and for patients with IL-8 > 62 pg/mL, more attention is needed to avoid the disease progression. Based on clinical practice and ROC analysis between critical and non-critical patients, some cut-off values of the test items were obtained, and the cut-off values of IL2R and CRP had relatively high sensitivity. With IL2R > 793.5 U/mL or CRP > 30.7 ng/mL, progress to critical illness should be closely observed and prevented; while with IL2R > 621.5 U/mL or CRP > 6.2 ng/mL, progress to severe condition should be closely evaluated.

Preventing recognition and blocking the occurrence of inflammatory response, or new drugs development on immune regulation might be new breakthroughs in the control of COVID-19. Pathological examination found lymphocyte-dominated mononuclear cell infiltration in interstitial pulmonary tissue. CD4+ and CD8+ T cells were significantly reduced in peripheral blood cells, but a high ratio of CD38+ (CD4 3.47%) and HLA-DR+ (CD8 39.4%) T cells [9], which suggested excessive activation of pro-inflammatory cells. Presumably, lymphocyte deposition in lung tissue might contribute to lymphocyte reduction in peripheral blood. In addition, researchers found that CCR4+ CCR6+ Th17 cells were increased, and the IL-17 inhibitor (Secukinumab) against activated Th17 cells might be promising for disease control [10]. In this study, IL-6, TNFα and IL-8 might be potential targets for immunotherapy of COVID-19. With IL-6 > 100 pg/mL, the patient’s condition was extremely critical. According to the news, a research team from First Affiliated Hospital of University of Science and Technology of China has used IL-6 receptor recombinant monoclonal antibody, Tozhu monoclonal antibody, in 14 critically or severely ill COVID-19 patients (https://mp.weixin.qq.com/s/b8v40pNb1H3TkzlOeLoysw, 2020/2/23), and the results were reported to be encouraging. Respiratory oxygenation indices of 14 patients have improved at different degrees, suggesting the potential of blocking inflammatory progression to reduce mortality (https://mp.weixin.qq.com/s/b8v40pNb1H3TkzlOeLoysw, 2020/2/23). IL-10 and IL2R levels were also related to disease severity, but they mainly inhibit the inflammatory response. Does it mean the simultaneous inflammatory and the anti-inflammatory reaction? The role of immunosuppression in disease progression and whether IL-10 and IL2R are possible therapeutic targets remains to be studied.

No significant difference was found about reduction ratio of B lymphocyte in the Wan S’s study [7]. In this study, there was no significant difference about Ig A, Ig G, IgM, C3 and C4 concentrations. The role of humoral immunity in the recovery of COVID-19 needs further study.

Severe bacterial, fungal and parasitic infections, sepsis, systemic inflammatory response syndrome, as well as multiple organ dysfunction syndrome and PCT levels in serum are elevated, while PCT is generally not elevated with virus infections [11]. In our study, PCT concentrations in all critical patients were > 0.05 ng/mL, and it suggested the possibility of multiple infections in critically ill patients and the necessity of rational use of antibiotics. With elevated WBC and PCT, multiple infections may occur; without increased PCT, the increase of WBC and NC might be induced by glucocorticoids. In clinical practice, few young and middle-aged women without basic disease progressed to critically illness, but our study did not find a gender correlation. Besides, No significant difference in ESR was found among three groups, which is different from Chen N’s study [7].

The purpose of this study was to analyze the relation between inflammatory indicators and critical illness. Some information was not included in the study, such as underlying diseases, course of disease and medication. Besides, glucocorticoids have effects on WBC and NC counts, and the treatment procedure was not cinsidered in the analysis, which means the statistical differences of WBC and NC need further verification. Another defect of the study is the limited sample size, and the conclusion needs to be further supported. Data collection and analysis were completed at the early stage of the epidemic, conclusions need to be verified with a longer period of surveillance. In the next study, more sample size, combined analysis with basic diseases, coagulation function, myocardial enzymes, liver and kidney function, and the type, dosage and time of medication are needed to identify the critical disease progression factors and better medication.

## Conclusions

Inflammation is closely related to severity of COVID-19. With increased inflammatory parameters, close attention should be paid to possible changes of disease severity, and IL-6 and TNFα might be promising therapeutic targets.

## Supplementary Information

**Additional file 1: Figure S1.** ROC curve of age and inflammatory parameters for severe illness of COVID-19. (A) age; (B) IL2R; (C) CRP; (D) ferroprotein; (E) WBC; (F) NC. IL2R, interleukin-2 receptor; CRP, C-reactive protein; WBC, white cell counts; NC, neutrophil count. AUC, 95% CI and *P* values are shown in the figure.

**Additional file 2: Table S1.** Reference range and analytical levels of inflammation-related test items. **Table S2.** Correlation coefficient and *P* value between items and disease severity. **Table S3.** Logistic regression analysis of inflammatory parameters and disease severity.

## Data Availability

The datasets used and/or analysed during the current study are available from the corresponding author on reasonable request.

## Abbreviations

IL2R: Interleukin-2 receptor
IL-6: Intereukin-6
IL-8: Interleukin-8
IL-10: Interleukin-10
TNFα: Tumor necrosis factor α
CRP: C-reactive protein
PCT: Procalcitonin
WBC: White cell counts
LC: Lymphocyte counts
NC: Neutrophil count
EC: Eosinophil counts
