# Supplementary figures and images for: Correlation analysis between disease severity and inflammation-related parameters in patients with COVID-19: a retrospective study
Source: BMC Infect Dis. 2020 Dec 21;20:963. doi: 10.1186/s12879-020-05681-5 (PMC7750784; doi:10.1186/s12879-020-05681-5)

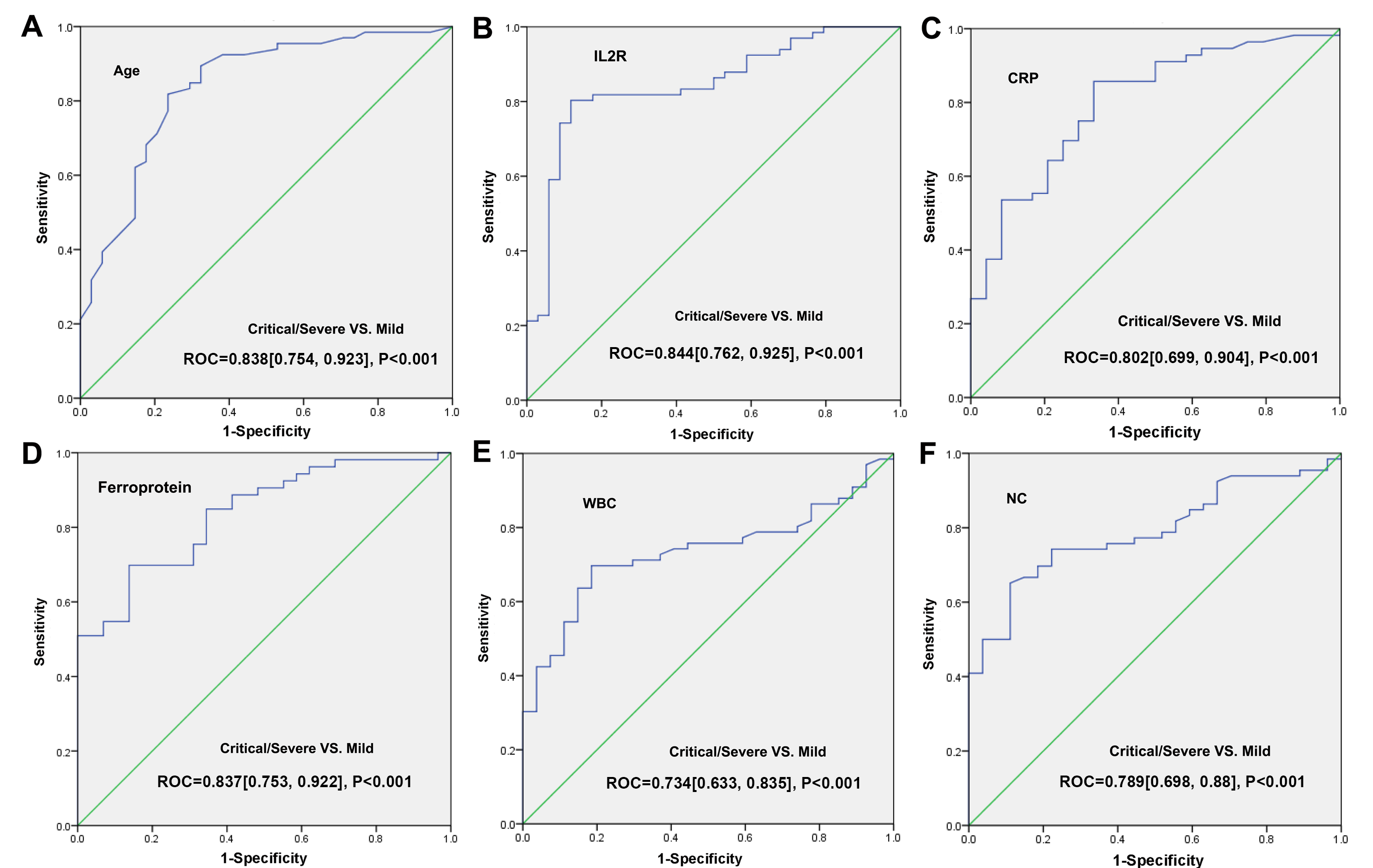

Supplement: Supplementary file 1 — Additional file 1: Figure S1. ROC curve of age and inflammatory parameters for severe illness of COVID-19. (A) age; (B) IL2R; (C) CRP; (D) ferroprotein; (E) WBC; (F) NC. IL2R, interleukin-2 receptor; CRP, C-reactive protein; WBC, white cell counts; NC, neutrophil count. AUC, 95% CI and P values are shown in the figure. [file 12879_2020_5681_MOESM1_ESM.tif]
