# Supplementary material for: Correlation analysis between disease severity and inflammation-related parameters in patients with COVID-19: a retrospective study
Source: BMC Infect Dis. 2020 Dec 21;20:963. doi: 10.1186/s12879-020-05681-5 (PMC7750784; doi:10.1186/s12879-020-05681-5)
Supplement: Supplementary file 2 — Additional file 2: Table S1. Reference range and analytical levels of inflammation-related test items. Table S2. Correlation coefficient and P value between items and disease severity. Table S3. Logistic regression analysis of inflammatory parameters and disease severity. [file 12879_2020_5681_MOESM2_ESM.doc]

Table S1. Reference range and analytical levels of inflammation-related test items.

| **Test item** | **Reference range** | **analytical levels** | | | |
| --- | --- | --- | --- | --- | --- |
|  |  | 1 | 2 | 3 | 4 |
| **IL-2R** | 223-710 (U/mL) | - | | | |
| **IL-6** | <7 (pg/mL) | <7 | 7-30 | 31-100 | >100 |
| **IL-8** | <62 (pg/mL) | 0-31 | 31-62 | >62 | - |
| **IL-10** | <9.1 (pg/mL) | ＜5 | 5-9.1 | 9.1-20 | >20 |
| **TNF-α** | <8.1 (pg/mL) | <8.1 | 8.1-11 | >11 | - |
| **IL-1β** | <5 (pg/mL) | <5 | ≥5 | 1 | |
| **CRP** | <1mg/L | 1 | | | |
| **Ferroprotein** | Male: 30-400 (μg/mL)  Female:15-150(μg/mL) | - | | | |
| **Procalcitonin** | 0.02-0.05ng/mL | <0.05 | 0.05-0.1 | 0.1-1 | >1 |
| **ESR** | 0-20mm/H | - | | | |
| **WBC** | 3.5-9.5*10^9/L | - | | | |
| **NC** | 1.8-6.3*10^9/L | - | | | |
| **LC** | 1.1-3.2*10^9/L | - | | | |
| **EC** | 0.02-0.05*10^9/L | >0.1 | 0.02-0.1 | 0-0.02 | 0 |

Table S2. Correlation coefficient and P value between items and disease severity.

|  | **R** | **P** |
| --- | --- | --- |
| **age** | -0.564 | 0.000 |
| **gender** | 1.0 | 0.291 |
| **IL-2R** | -0.534 | 0.000 |
| **IL-6** | -0.535 | 0.000 |
| **IL-8** | -0.308 | 0.01 |
| **IL-10** | -0.422 | 0.000 |
| **TNF-α** | -0.322 | 0.000 |
| **IL-1β** | 0.098 | 0.301 |
| **CRP** | -0.604 | 0.000 |
| **Ferroprotein** | -0.508 | 0.000 |
| **Procalcitonin** | -0.650 | 0.000 |
| **ESR** | -0.261 | 0.061 |
| **WBC** | -0.54 | 0.000 |
| **NC** | -0.585 | 0.000 |
| **LC** | 0.56 | 0.000 |
| **EC** | 0.299 | 0.01 |

Table S3. Logistic regression analysis of inflammatory parameters and disease severity.

| **Variable** | **term** | **estimate** | **std.error** | **statistic** | **OR[95%CI]** | **p.value** |
| --- | --- | --- | --- | --- | --- | --- |
| **(Intercept)** | (Intercept) | -10.99 | 3.28 | -3.35 | 0.00 [0.00, 0.00] | 0.001 |
| **sexfemale** | sexfemale | 1.73 | 1.16 | 1.49 | 5.64 [0.71, 80.99] | 0.136 |
| **`IL-1β`2** | `IL-1β`2 | -3.88 | 1.97 | -1.97 | 0.02 [0.00, 0.50] | 0.049 |
| **`IL-6`2** | `IL-6`2 | 3.49 | 1.44 | 2.43 | 32.80 [2.77, 1034.58] | 0.015 |
| **`IL-6`3** | `IL-6`3 | 2.45 | 1.49 | 1.64 | 11.60 [0.84, 397.93] | 0.101 |
| **`IL-6`4** | `IL-6`4 | 5.27 | 2.37 | 2.22 | 193.57 [4.22, 78209.86] | 0.027 |
| **`IL-8`2** | `IL-8`2 | -2.62 | 1.46 | -1.80 | 0.07 [0.00, 0.91] | 0.072 |
| **`IL-8`3** | `IL-8`3 | 17.54 | 1802.40 | 0.01 | 41620321.67 [0.00, NA] | 0.992 |
| **`IL-10`2** | `IL-10`2 | 1.83 | 1.18 | 1.54 | 6.21 [0.63, 76.81] | 0.123 |
| **`IL-10`3** | `IL-10`3 | 2.85 | 1.24 | 2.30 | 17.33 [1.90, 278.21] | 0.021 |
| **`IL-10`4** | `IL-10`4 | -0.80 | 1.59 | -0.50 | 0.45 [0.01, 8.21] | 0.614 |
| **WBC** | WBC | 3.00 | 1.33 | 2.25 | 20.04 [2.09, 422.73] | 0.025 |
| **Lymphocyte** | Lymphocyte | -3.42 | 1.48 | -2.31 | 0.03 [0.00, 0.39] | 0.021 |
